# Cardiovascular Complications of COVID-19 Vaccines

**DOI:** 10.3389/fcvm.2022.840929

**Published:** 2022-03-18

**Authors:** Runyu Liu, Junbing Pan, Chunxiang Zhang, Xiaolei Sun

**Affiliations:** ^1^Department of General Surgery (Vascular Surgery), The Affiliated Hospital of Southwest Medical University, Luzhou, China; ^2^Key Laboratory of Medical Electrophysiology, Ministry of Education and Medical Electrophysiological Key Laboratory of Sichuan Province, Collaborative Innovation Center for Prevention and Treatment of Cardiovascular Disease of Sichuan Province, Institute of Cardiovascular Research, Southwest Medical University, Luzhou, China; ^3^Cardiovascular and Metabolic Diseases Key Laboratory of Luzhou, Luzhou, China; ^4^Nucleic Acid Medicine of Luzhou Key Laboratory, Southwest Medical University, Luzhou, China; ^5^Department of Interventional Medicine, The Affiliated Hospital of Southwest Medical University, Luzhou, China; ^6^King's College London British Heart Foundation Centre of Research Excellence, School of Cardiovascular Medicine and Sciences, Faculty of Life Science and Medicine, King's College London, London, United Kingdom

**Keywords:** COVID-19, vaccine, cardiovascular, complication, mRNA

## Abstract

Coronavirus disease 2019 (COVID-19) has become a global public health catastrophe. Vaccination against severe acute respiratory syndrome coronavirus-2 (SARS-CoV-2) is proven to be the most effective measure to suppress the pandemic. With the widespread application of the four vaccines, namely, ChAdOx1, Ad26.COV2.S, BNT162b2, and mRNA-1273.2, several adverse effects have been reported. The most serious type of complication is cardiovascularly related, including myocarditis, immune thrombocytopenia (ITP), cerebral sinus venous thrombosis, among others. All these adverse events undermine the health of the vaccinees and affect the administration of the vaccines. As the distribution of COVID-19 vaccines is surrounded by suspicion and rumors, it is essential to provide the public with accurate reports from trusted experts and journals. Monitoring the safety of COVID-19 vaccines is an important and ongoing process that is also urgent. Thus, we summarized the cardiovascular complications of the major types of COVID-19 vaccines, including mRNA vaccines, which are now generally considered to be innovative vaccines, and the future for vaccination against COVID-19, in addition to the underlying pathogenesis and potential therapeutics.

## Introduction

**Figure 1:**
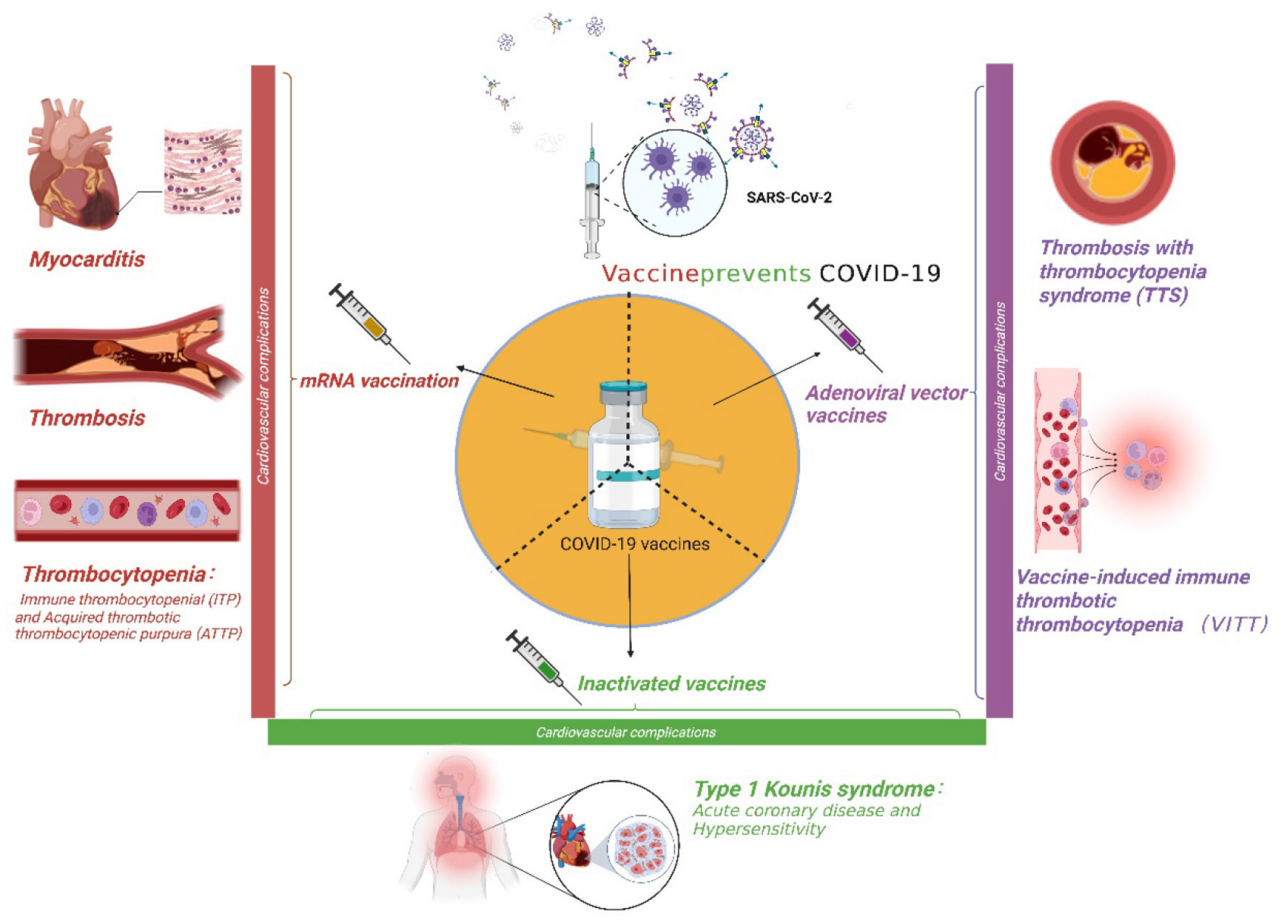
The cardiovascular complications of three major coronavirus disease 2019 (COVID-19) vaccines.

Severe acute respiratory syndrome coronavirus-2 (SARS-CoV-2), also known as coronavirus disease 2019 (COVID-19), has spread rapidly throughout the world, leading to acute respiratory distress syndrome (ARDS). COVID-19 has become a global public health catastrophe. Vaccination against SARS-CoV-2 is now proven to be the most effective means of suppressing the pandemic (1). COVID-19 vaccines showed high efficacy against SARS-CoV-2 during the different phases of clinical trials (1). The first four vaccine preparations (i.e., ChAdOx1 and AD26.COV2·S, BNT162b2, and mRNA-1273) have received marketing authorization from the European Medicines Agency (EMA) (2). With the widespread application of these four vaccines, several adverse effects, such as pain at the site of inoculation, fever, and allergic reactions, have been reported (3). The most serious complications are cardiovascularly related, and these complications include myocarditis, immune thrombocytopenia (ITP), cerebral sinus venous thrombosis, and visceral thrombosis. All of these adverse events impair the health of those receiving vaccinations and affect the administration of the vaccines (4, 5). Thus, it is necessary to summarize the cardiovascular complications of the major types of COVID-19 vaccines (Figure 1) and review the underlying pathogenesis and potential therapies.

### mRNA Vaccines

At present, there are two types of mRNA vaccines used to prevent SARS-CoV-2, namely, Pfizer's BNT162b2 mRNA vaccine and Moderna's mRNA-1273 vaccine. mRNA vaccines use the mRNA that encodes the spike protein of SARS-CoV-2, surrounded by lipid nanoparticles (LNPs). The spike protein induces the body to produce the corresponding antibodies, which causes recipients to develop immunity to SARS-CoV-2. The BNT162b2 mRNA vaccine has been reported to be effective in a variety of COVID-19 clinical trials (6). However, the development of cardiovascular adverse events after the administration of these mRNA vaccines should be seriously considered.

#### Myocarditis After COVID-19 mRNA Vaccination

A total of 561,197 people in North Carolina were vaccinated from February 1 to April 30, 2021. Later, the Duke University Medical Center in Durham reported 4 cases of myocarditis; in all cases, the patients developed severe chest pain with biomarker evidence of myocardial damage, and all 4 patients were later hospitalized (4). In another case, one Filipino patient was diagnosed with myocarditis 3 days after receiving the second dose of the BNT162b2 mRNA vaccine (7). From January 30 to February 20, 2021, six patients with chest pain were treated in the Hillel Yaffe Medical Center, Israel, soon after vaccination with the BNT162b2 mRNA vaccine. All six of these patients were diagnosed with myocarditis, and one of these patients had received only the first dose of the vaccination (8). As mRNA vaccination becomes more widespread, an increasing number of myocarditis cases have been diagnosed. All patients with mild symptoms can be discharged within 4–8 days after treatment with non-steroidal anti-inflammatory drugs or colchicine (8). As on June 11, 2021, more than 296 million doses of COVID-19 mRNA vaccine had been administered in the United States, of which 52 million were administered to people aged 12–29 years. From December 29, 2020, to June 11, 2021, the Vaccine Adverse Event Reporting System (VAERS) received 1,226 reports of myocarditis after mRNA vaccination. The risk of myocarditis increases within 7 days after the first or second dose of an mRNA vaccine (9). The Centers for Disease Control and Prevention reported two cases of histologically confirmed myocarditis after COVID-19 mRNA vaccination on August 18, 2021. A 42-year-old man developed breathing difficulties and chest pain 2 weeks after receiving the second dose of the mRNA-1273 vaccine. It was also reported that he had no viral prodrome, and his PCR tests were negative for SARS-CoV-2. The patient developed tachycardia and fever. An electrocardiogram (ECG) showed diffuse ST-segment elevation, and Doppler echocardiography showed biventricular dysfunction (ejection fraction, 15%). The patient died of cardiogenic shock 3 days after the visit, and an autopsy revealed biventricular myocarditis (10). The potential mechanisms of mRNA vaccine-induced myocarditis are still unclear. It has been reported that the mRNA-1273 vaccine can induce a strong CD4 cytokine response involving type 1 helper T (Th1) cells, and CD4 cells are an important factor in myocarditis (11, 12). The detailed mechanisms warrant further clinical and basic research.

#### Thrombosis With COVID-19 mRNA Vaccination

The Medicines and Healthcare Products Regulatory Agency in the United Kingdom reported that, with the administration of 10.6 million doses of the BNT162b2 mRNA vaccine, there were 24 cases of cerebral venous sinus thrombosis (CVST), 3 cases of cerebral vascular thrombosis, 3 cases of superior sagittal sinus thrombosis, and 1 case of transverse sinus thrombosis (13). Among 4 million doses of mRNA-1273 that have been administered, 5 cases of suspected CVST have been reported (14). The mechanism underlying the association between mRNA vaccination and thrombosis is unelucidated. It is speculated that it is related to the encoding of the SARS-CoV-2 spike protein by mRNA vaccines. The spike protein, which is necessary to allow SARS-CoV-2 to invade human cells, enhances platelet aggregation and promotes the secretion of dense granules from platelets (15). Moreover, as a binding ligand of the ACE2 receptor, the SARS-CoV-2 spike protein induces an inflammatory response in brain endothelial cells and impairs the functional integrity of the blood-brain barrier, which promotes the activation of endothelial cells and the upregulation of leucocyte chemokines, pro-inflammatory cytokines (interleukin (IL)-1β and IL-6), and cell adhesion molecules [intercellular adhesion molecule 1 (ICAM-1) and vascular cell adhesion molecule 1 (VCAM-1)] (16). All of these mRNA vaccine-related pathophysiological activities might initiate the development of thrombosis. Thus, anti-spike protein monoclonal antibodies or recombinant human ACE2 proteins might assist in the treatment of patients with COVID-19 mRNA vaccine-induced thrombosis (15). However, at present, the current clinical treatment of choice is anticoagulation therapy with unfractionated heparin, followed by low-molecular-weight heparin and then warfarin (13).

#### Vaccine-Induced Thrombocytopenia

One day after receiving the first dose of the mRNA-1273 vaccine, a 60-year-old African-American man developed the symptoms of low-grade fever and chills, followed by the appearance of a severe generalized rash on his skin that quickly spread throughout his body. In addition, he was diagnosed with ITP (17). From mid-February to mid-March, nearly 5 million people in Israel were vaccinated with the BNT162b2 mRNA vaccine, and four patients were diagnosed with acquired thrombotic thrombocytopenic purpura (ATTP) (18). In the United States, more than 20 million people (as on February 2, 2021) have received at least one dose of either of the two available mRNA vaccines, and 20 of these patients developed thrombocytopenia after vaccination. Most of the 20 patients had symptoms, such as bruises or mucosal bleeding. Nine of these patients were vaccinated with the BNT162b2 mRNA vaccine, and 11 of these patients were vaccinated with the mRNA-1273 vaccine (19). Interestingly, in the United States, approximately 50,000 adults are diagnosed with ITP each year. Thus, the incidence rate of mRNA vaccine-related ITP is almost the same as that of the baseline incidence rate for the population (19). However, most of these patients developed ITP symptoms after their first dose of COVID-19 mRNA vaccination; therefore, there seems to be a link between ITP and mRNA vaccine administration. However, the potential mechanism underlying the relationship between mRNA vaccines and ITP is unknown. Vaccines can activate autoimmunity through molecular mimicry, which induces the production of antiplatelet autoantibodies and causes thrombocytopenia (20).

## Adenoviral Vector vaccines

At present, the cardiovascular complications that have been observed in association with adenoviral vector vaccines, i.e., the ChAdOx1 nCoV-19 (Oxford-AstraZeneca [AZ]; also known as Vaxzevria) vaccine and AD26.COV2·S (Johnson & Johnson [JJ]) vaccine, are primarily associated with thrombosis with thrombocytopenia syndrome (TTS). The ChAdOx1 nCoV-19 and AD26.COV2·S vaccines are composed of recombinant adenovirus vectors from chimpanzee adenovirus or human adenovirus, which encode the spike protein of SARS-CoV-2 (21). As on April 7, 2021, 34 million people had been vaccinated with ChAdOx1 in the European Economic Area and the United Kingdom, and the EMA reported 169 cases of cerebral venous thrombosis and 53 cases of splanchnic vein thrombosis (SVT) after vaccination (2). Six cases of suspected CVST have been reported among more than 7 million recipients of the AD26.COV2·S adenovirus vector vaccine (14). In the context of a worldwide vaccination campaign, these safety issues should not be ignored. Most patients with thrombosis are positive for anti-PF4 antibodies, which have effects similar to heparin-induced thrombocytopenia (HIT); therefore, this syndrome was named vaccine-induced immune thrombotic thrombocytopenia (VITT) (2, 14). VITT mainly occurs in women under the age of 55 years, often occurs 4–16 days after patients receive an adenovirus-based vaccine, and is associated with a high mortality rate (14, 22). A 35-year-old pregnant woman developed intracerebral hemorrhage in the left temporal lobe associated with VITT 12 days after off-label ChAdOx1 nCoV-19 vaccination. This pregnant woman, who was at 23 weeks of gestation, died on day 17 (after vaccination) of refractory intracranial hypertension despite the use of all available pressure control measures (23).

The activation and depletion of platelets in VITT do not rely on heparin (2, 21, 24). Why, then, do anti-PF4 antibodies appear? It has been reported that the presence of PF4-immunoglobulin G (IgG) antibodies increases with the severity of trauma (25); therefore, the production of PF4 antibodies may be associated with an inflammatory response following adenovirus vector vaccination. As PF4 is released by platelets and forms a complex with heparin in the pathogenesis of HIT, the human body forms an IgG against the PF4-heparin complex. Another hypothesis is that after vaccination, viral proteins and free DNA bind to PF4 and form a new antigen (26). These antibodies can bind to FcγRIIa on platelets, promoting platelet activation and aggregation and thus leading to thrombosis (27). In addition, the activation of von Willebrand factor (vWF) and P-selectin after the administration of adenoviral vector vaccines plays a key role in complexes of platelets, white blood cells, and endothelial cells and accelerates the activation and clearance of platelets (28, 29). The other underlying mechanism is that the ethylenediaminetetraacetic acid (EDTA) in vaccine preparations may increase vascular permeability at the injection site and may cause the vaccine components to spread through the bloodstream, which may produce a signal that leads to the production of anti-PF4 antibodies in B cells (2).

However, not all adenoviral vector vaccines induce similar symptoms. No VITT-related adverse events have been reported for the AD5 adenovirus vector vaccine produced by CanSino Biologics (2). The principles for the treatment of VITT are the administration of intravenous immunoglobulin, anticoagulation, the avoidance of heparin, and the transfusion of platelets (30). If the platelet levels are >30 × 10^9^/L and if fibrinogen is >1.5 g/L, non-heparin anticoagulation, including argatroban, bivalirudin, apixaban, or rivaroxaban, is suggested (20).

## Inactivated Vaccines

Inactivated vaccines have been extensively studied and have the advantage of being easy to store and transport, making them suitable for many low-income countries (31). The BBIP-CorV inactivated vaccine was produced by Sinopharm in China and developed from the HB02 strain isolated from patients at the Jinyintan Hospital in Wuhan, China. The ZhongkangKewei (WIBP-CorV) inactivated vaccine, developed from the WIVO4 strain, was also isolated from patients at the Jinyintan Hospital (31). The CoronaVac vaccine is produced by Sinovac Life Sciences in China. In the 40,382 recipients of these vaccines, all three inactivated vaccines had a high level of safety and efficacy (>70%), and none of the patients developed serious cardiovascular adverse events related to vaccination (31). However, in Turkey, among more than 7 million vaccinated people, a 41-year-old woman without any cardiovascular risk factors developed symptoms that included facial flushing, chest palpitations, and chest pain 15 min after receiving the first vaccine dose. She was diagnosed with type one Kounis syndrome, which is a combination of acute coronary disease and hypersensitivity. After treatment with oral antihistamine and aspirin, the patient improved and was discharged from the hospital (32). Although this is the first reported case of allergic myocardial infarction secondary to the administration of an inactivated vaccine, more clinical data and research on the underlying mechanism are needed.

The cardiovascular complications associated with COVID-19 vaccines are presented in detail in Table 1.

**Table 1:** Cardiovascular complications of coronavirus disease 2019 (COVID-19) vaccines.

**Vaccine**	**Data source or region**	**Time**	**Complication**	**Total number administered**	**Number of events**	**Complication rate**
BNT162B2 (8)	Israel	To 2021.3.24	Myocarditis	More than 4 million	6	1.5/1 million
BNT162B2 (13)	MHRA	2020.12.9–2021.5.26	Thrombosis	10.6 million	33	3.11/1 million
BNT162B2 (13)	Singapore	To 2021.5.31	Thrombosis	1,766,493	3	1.70/1 million
BNT162B2 (14)	EMA	Unknown	Thrombosis	54 million	35	0.65/1 million
mRNA vaccine (19)	VAERS	To 2021.2.2	ITP	20 million	20	1.00/1 million
BNT162B2 (18)	Israel	2021.2–2021.3	ATTP	5 million	4	0.80/1 million
mRNA vaccine (4)	DUMC	2021.2.1–2021.4.30	Myocarditis	561,197	4	7.14/1 million
mRNA vaccine (9)	USA	2020.12.29–2021.6.1	Myocarditis	52 million	1,226	23.58/1 million
mRNA-1273 (14)	EMA	Unknown	Thrombosis	4 million	5	1.25/1 million
mRNA vaccine and ChAdOx1 (13)	VigiBase	2020.12.12–2021.3.16	Thrombosis	Unknown	2,169	Unknown
ChAdOx1 (1)	PRAC	To 2021.4.7	Thrombotic thrombocytopenia	34 million	222	6.53/1 million
ChAdOx1 (21)	UK	To 2021.4.14	Thrombotic thrombocytopenia	21.2 million	168	7.92/1 million
AD26.COV2·S (21)	USA	To 2021.4.13	Thrombosis	6.8 million	15	2.21/1 million
AD26.COV2·S (14)	EMA	Unknown	Thrombosis	More than 7 million	6	0.86/1 million

*MHRA, Medicines and Healthcare Products Regulatory Agency of the United Kingdom; EMA, European Medicines Agency; VAERS, Vaccine Adverse Events Reporting System; DUMC, Duke University Medical Center; VigiBase, WHO Global Database for Individual Case Safety Reports; PRAC, the EMA's Pharmacovigilance Risk Assessment Committee*.

## Discussion

The emergence of the delta variant (Pango lineage B.1.617.2) of SARS-CoV-2 has caused a global resurgence of the pandemic. However, fortunately, the latest real-world data have revealed that COVID-19 vaccines, (33) especially the BNT162b2 mRNA vaccine, still have 88% efficacy for preventing the symptomatic morbidity of the delta variant of SARS-CoV-2 (34). With the increasingly widespread use of COVID-19 vaccines, safety issues associated with the vaccines are gradually becoming the focus of public concern.

Inactivated vaccines have been used for many years to prevent a variety of infectious diseases, and consequently, their safety is generally considered good. However, cardiovascular-related allergic events can occur during vaccination. According to the available literature, the frequency of severe allergic reactions after inactivated vaccine administration appears to be low. However, type one Kounis syndrome is one such rare serious adverse event. Physicians should be aware that Kounis syndrome is a rare but dangerous complication of inactivated coronavirus vaccines. Patients who develop chest pain or severe allergic reactions after vaccination should undergo ECG, echocardiography, and troponin measurement, and these patients should undergo adequate observation or hospitalization if necessary (32).

The VITT, a particularly rare cardiovascular complication, has been observed in adenoviral vector vaccines but not in the other types of vaccines; however, cases of thrombocytopenia after mRNA vaccination have been reported and may be due to an autoimmune mechanism. The key point is whether the VITT/TTS was observed with ChAdOx1 nCoV-19 and AD26.COV2·S vaccinations to represent side effects specific to adenovirus vector vaccines and the extent to which this may affect the administration of the adenoviral vector vaccines. There have been reports suggesting that the incidence of VITT/TTS may be much higher than previously assumed, and this incidence may further increase as physicians become increasingly aware of the syndrome. The estimated incidence of VITT varies among different reports, from ~1 in 25,000 vaccinated with ChAdOx1 and 1 in more than 500,000 vaccinated with AD26.COV2·S (2). However, these findings should not be used as a reason to discontinue the use of ChAdOx1 nCoV-19 or AD26.COV2·S vaccines. The incidence of VITT complications following adenovirus vector vaccination remains low, while the COVID-19 infection rate and mortality rate are much higher (25).

The mRNA vaccines are innovative vaccines that represent the future of vaccination against COVID-19, and they have the advantages of low production costs and short production cycles. Although the BNT162b2 and mRNA-1273 vaccines were 95% effective after two doses in a phase III clinical trial, (35) the long-term efficacy of these mRNA vaccines is poorly understood. Although mRNA vaccines have been proven to be highly preventive, their cardiovascular side effects should also be seriously considered. Acute myocarditis is a critical adverse event after mRNA vaccination, especially in young males, and these adverse events should be considered in patients who develop cardiac symptoms after receiving an mRNA vaccine (7). However, the specific mechanism needs to be further explored in larger studies. The cases of mRNA vaccine-secondary ITP following the administration of the BNT162b2 mRNA vaccine or the mRNA-1273 vaccine have been reported and have raised public concern (17, 18). Public panic intensified after the first confirmation of a patient who died of an intracranial hemorrhage (19). The incidence of ITP after mRNA vaccination was actually not far from the estimated baseline annual incidence in the general population; however, post-vaccination ITP remains a possibility, especially in patients with an onset of 1–2 weeks after exposure (19). For patients with cardiovascular complications after mRNA vaccination, such as myocarditis, thrombosis, and ITP, further study is needed to determine whether a second dose of vaccine is needed, whether a different type of vaccine should be used, or whether ITP following the initial dose will exacerbate all of these problems. British researchers launched a phase I clinical trial of a second-generation COVID-19 vaccine on September 20, 2021. The new vaccine, named GRT-R910, (36) is a self-amplified mRNA vaccine. With the global trend of the development of new mRNA vaccines, it is important for researchers to pay increased attention to these possible fatal cardiovascular complications.

The attitudes of people in the community toward vaccination against COVID-19 have been volatile since the end of 2019. People were reluctant to be vaccinated at the initial stage of the application of the new COVID-19 vaccines, as the mid- and long-term data were lacking. As the pandemic worsened in 2020, many people were in favor of being vaccinated. However, as the number of adverse events, especially cardiovascular complications, of these COVID-19 vaccines merged, people became conflicted about whether to be vaccinated, even when the delta variant and the subsequent omicron variant merged. In contrast, due to a lack of scientific and prompt information and data, people are concerned about the possible complications of these vaccines and refuse to allow themselves or their children to be vaccinated. This contradiction also impacts their normal life, resulting in different degrees of anxiety (37). For countries around the world, spending on nationwide COVID-19 vaccination under the suspicion of these adverse events might have unprecedented budget implications for governments and commercial payers. Governments should focus on expanding health system infrastructure and subsidize payer coverage to deliver these vaccines effectively (38). Since the outbreak of the pandemic in 2019, people have been concerned about the complications of vaccines, which have sparked anti-vaccine movements. It is now most important to raise public awareness of COVID-19 vaccine complications through urgent education to reduce the negative impact of a lack of knowledge of COVID-19 vaccination decisions (39).

Although multiple COVID-19 vaccine-related cardiovascular adverse events have been reported, vaccines are still widely used because they are effective against the virus. Compared with the low incidence of complications, the high efficacy of the vaccines against COVID-19 suggests that COVID-19 vaccines should be widely administered. In fact, the cardiovascular complications caused by vaccines can be effectively treated, and most patients improve quickly. Furthermore, a recent study indicated that SARS-CoV-2 infection is itself a very strong risk factor for myocarditis, and the virus also substantially increases the risk of many other serious adverse events (40). A syndrome called Long-COVID-19 has recently emerged among COVID-19 survivors, which is characterized by persistent, typical acute symptoms accompanied by changes in inflammatory and coagulation parameters caused by endothelial damage. SARS-CoV-2 causes the activation of local and circulating coagulation factors, inducing the production of diffuse coagulation. The similarities and differences between the cardiovascular complications caused by COVID-19 and those caused by mRNA vaccines *via* the spike protein need to be further studied (41). Thus, people should not refuse vaccinations or promote conspiracy theories out of fear of vaccine-related complications.

As the distribution of COVID-19 vaccines is surrounded by suspicion and rumors, it is essential to provide the public with accurate reports from trusted experts, such as medical professionals. Monitoring the safety of COVID-19 vaccines is an important and ongoing process that warrants urgent attention. We propose the establishment of a global database on COVID-19 vaccine adverse events to collect precise and continuous data. In addition, regional regulatory systems should regulate vaccine administration and monitor the occurrence of adverse events and their follow-up in vaccinated people.

## Author Contributions

All authors listed have made a substantial, direct, and intellectual contribution to the work and approved it for publication.

## Funding

This study was supported by the Collaborative Innovation Center for Prevention and Treatment of Cardiovascular Disease of Sichuan Province Funded Project (Grant code: xtcx2019-20), Key Laboratory of Medical Electrophysiology of Ministry of Education Funded Project (Grant code: KeyME-2018-04), Open Program of Nuclear Medicine and Molecular Imaging Key Laboratory of Sichuan Province (Grant code: HYX19007), and Doctoral Research Initiating Program of Affiliated Hospital of Southwest Medical University (Grant code: 19041).

## Conflict of Interest

The authors declare that the research was conducted in the absence of any commercial or financial relationships that could be construed as a potential conflict of interest.

## Publisher's Note

All claims expressed in this article are solely those of the authors and do not necessarily represent those of their affiliated organizations, or those of the publisher, the editors and the reviewers. Any product that may be evaluated in this article, or claim that may be made by its manufacturer, is not guaranteed or endorsed by the publisher.
